# High-Dose-Rate Three-Dimensional Image-Guided Adaptive Brachytherapy (3D IGABT) for Locally Advanced Cervical Cancer (LACC): A Narrative Review on Imaging Modality and Clinical Evidence

**DOI:** 10.3390/curroncol31010004

**Published:** 2023-12-21

**Authors:** Kaiyue Wang, Junjie Wang, Ping Jiang

**Affiliations:** Department of Radiation Oncology, Peking University Third Hospital, Beijing 100191, China; wngkyu@pku.edu.cn (K.W.); junjiewang@pku.edu.cn (J.W.)

**Keywords:** cervical cancer, image guidance, treatment planning, clinical outcomes, interstitial brachytherapy

## Abstract

**Background**: Brachytherapy (BT) is a critical component of radiotherapy for locally advanced cervical cancer (LACC), and it has rapidly developed in recent decades. Since the advent of three-dimensional image-guided adaptive brachytherapy (3D-IGABT), magnetic resonance imaging (MRI) has emerged as the primary modality for image guidance. Meanwhile, other imaging modalities, such as computed tomography, 18F-fluorodeoxyglucose positron emission tomography, ultrasound, and their combinations have also been widely studied. **Materials and methods**: We reviewed studies on different imaging modalities utilized for target delineation and planning. Emerging techniques in IGABT like real-time image guidance and 3D printing were also included. We summarized research on their feasibility and concentrated on their clinical outcomes. **Results**: MRI-guided BT was the gold standard, and CT-guided BT was the most widely applied. Other modalities have shown feasibility and promising efficacy in dosimetry studies and preliminary outcomes. The longer-term clinical outcomes associated with these approaches require further elucidation. **Conclusions**: As 3D-IGABT was validated by promising clinical outcomes, the future of BT for LACC is expected to progress toward the refinement of more effective image-guided procedures. Moreover, achieving operational consensus and driving technological advancements to mitigate the inherent limitations associated with different imaging modes remain essential.

## 1. Introduction

Cervical cancer is the most frequent gynecological cancer worldwide, resulting in more than 340,000 deaths in 2020 [1]. Locally advanced cervical cancer (LACC) is specifically characterized by the inclusion of FIGO 2018 stages IB3 to IVA, accounting for more than 70% of newly diagnosed patients. The standard treatment choice for patients with LACC is concurrent chemoradiotherapy (CCRT), consisting of intracavity brachytherapy (ICBT) or interstitial brachytherapy (ISBT) following external-beam radiotherapy (EBRT). [2] Of note, brachytherapy (BT) is regarded as an irreplaceable radiotherapy to improve local control (LC) and overall survival (OS), which is characterized by steep dose gradients to enable a high dose boost while protecting organs at risk (OARs) [3].

Historically, two-dimensional (2D) orthogonal X-ray images have been used to define applicators and uterine locations. Point A was prescribed as a surrogate for tumor dose to achieve a pear-shaped isodose distribution [4,5]. Nonetheless, point-dose-based 2D BT inadequately represents the actual tumor dosage and OARs’ exposure [6,7], and it lacks the capacity to be adjusted in accordance with varying tumor sizes and locations [8]. Image-guided technology has enabled oncologists to visualize and contour the tumor (as well as normal tissues) on volumetric imaging, and optimize the dosimetry. The transition from a 2D Point A-based prescription to a three-dimensional (3D) volume-based dose prescription has been substantiated by existing evidence to confer advantages in terms of both survival rates and mitigation of toxicities [3,9,10,11]. Imaging modalities such as magnetic resonance imaging (MRI), computed tomography (CT), ultrasound (US), ^18^F fluorodeoxyglucose (FDG)-positron emission tomography (PET), and their combinations have also been widely studied. Apart from postimplant simulation images for contouring and planning, real-time imaging approaches are also used in ISBT to guide appropriate implants of the applicators and avoid repeated adjustments or suboptimal dose coverage.

This review aims to comprehensively overview advancements in the practice of modern 3D image-guided adaptive brachytherapy (3D IGABT), revealing the state-of-the-art landscape across the entire BT workflow, guided by diverse imaging techniques that are utilized for target delineation, planning, and real-time applicator guidance. We summarize research on their feasibility and clinical outcomes and provide perspectives on trends in 3D IGABT development.

## 2. MRI-Guided BT: The Gold Standard

### 2.1. MRI-Guided Contouring and Planning

**Figure 1:**
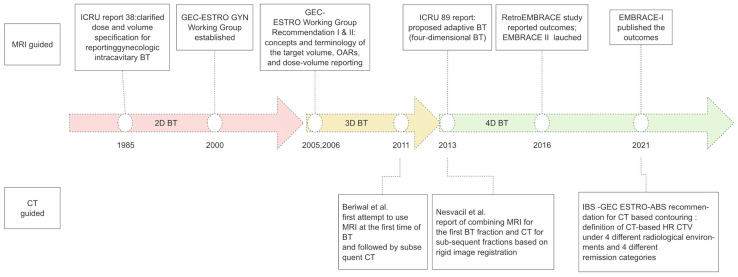
Milestones in brachytherapy for cervical cancer. A schematic timeline showing publications of note, including MRI-guided and CT-guided brachytherapy, which are the two most important imaging modalities [22,23].

As 3D-IGABT brachytherapy has become the mainstream treatment modality, MRI is the gold standard for IGABT due to its superior soft tissue contrast and ability to define parametrial and normal organ infiltrations [12,13,14]. Apart from BT, MR-guided radiotherapy (MRgRT), referring to MRI devices integrated with linear accelerators, so called “MR-linac”, is also currently under investigation in cancer EBRT [15,16]. To promote MRI-guided brachytherapy for gynecological tumors and achieve a common communication standard, the Groupe Européen de Curiethérapie (GEC) and the European Society for Radiotherapy & Oncology (ESTRO) (GEC-ESTRO) gynecological (GYN) Working Group was established in 2000, and published a series of guidelines to define the concepts and terminology of the definition of the target volume, OARs, and dose–volume reporting [10,17]. International Commission on Radiation Units and Measurements (ICRU) 89 guidelines [18] further proposed adaptive brachytherapy, requiring adjustment of the target volume and dose according to the changes in tumor shrinkage. Adaption of the time dimension regarding different patterns of treatment response is also referred to as 4D-IGABT [19]. For normal tissues, GEC-ESTRO guidelines [13] and the ICRU89 report [18] recommend delineating the outer walls of the OARs and reporting the minimum dose in the most exposed volume, such as D_0.1cc_ and D_2cc_. Meanwhile, EMBRACE serial studies launched and reported large-scale survival outcomes and morbidity patterns of MRI-based IGABT. These data provided necessary high-level evidence for dose prescriptions and predictors of local control [3,20]. For tumor target volume and OARs, dose–volume parameters and dose–effect relationships are further clarified. Risk factors and dose–effect relationships have opened up opportunities for tailored dose prescriptions, and further studies are expected to investigate dose escalation and de-escalation, as shown in Figure 1 [21]. 

### 2.2. Clinical Outcomes and Prognostic Factors

Due to the excellent visualization, the utilization of MRI guidance has demonstrated a substantial capacity to enhance tumor dose precision while simultaneously minimizing radiation exposure to OARs. Moreover, it has exhibited remarkable efficacy in terms of LC and survival outcomes [8]. The RetroEMBRACE study and several experiences from single-institution studies reported a satisfactory LC of 85–95% [3,24,25]. Subsequently, GEC-ESTRO published EMBRACE-I [20], a large-scale multicenter clinical study, which confirmed the superiority of MRI-guided BT in terms of efficacy and side effects. The 5-year LC reached 92% (92% CI 90–93%), the 5-year OS was 74% (72–77%), and the incidence of ≥G3 side effects was 14.6%.

From the analysis of clinical data, both tumor parameters and treatment factors were found to be associated with clinical outcomes. The RetroEMBRACE study findings indicated that factors such as tumor histology, CTV_HR_ D_90_ (the dose delivered to the high-risk clinical target volume), CTV_HR_ volume, and the overall treatment time (OTT) exerted a discernible influence on LC [26]. An analysis of the EMBRACE I study revealed that several factors played a significant role in LC. These factors included histology, CTV_HR_ D_90_, tumor size, OTT, the presence of tumor necrosis on MRI at diagnosis, and infiltration into the uterine corpus and mesorectal region [21]. The presence of tumor necrosis on MRI at the time of diagnosis, indicative of local hypoxia, along with other factors, such as the residual gross tumor volume (GTV_res_) during BT and the volume of the CTV_HR_, as well as the degree of tumor shrinkage during concurrent chemoradiotherapy, all hold significance in reflecting the tumor’s radiosensitivity and its potential impact on prognosis. Accordingly, Schernberg et al. [27] proposed employing a 90% reduction in GTV volume as a threshold for assessment. Ke et al. [28] demonstrated that a volume of > 8.37 cm^3^ GTV_res_ at the initial BT session (GTV_res BT1_) was associated with poor 2-year LC (84.0% vs. 98.5%, *p* = 0.025) and OS (52.0% vs. 89.7%, *p* < 0.01).

## 3. CT-Guided BT: The Most Common Procedure

Although MRI is optimal for 3D-IGABT planning in LACC, the routine utilization of MRI is not always readily accessible in some healthcare institutions due to logistical challenges and time-consuming image acquisition processes [29]. Furthermore, MRI can be susceptible to distortions and diminished image quality arising from organ movements during the procedure [30]. In contrast, CT presents promising prospects for development due to its excellent compatibility with BT applicators. Additionally, CT scans provide electronic voxel information, facilitating optimization in treatment planning. Several studies have shown that CT is the most commonly used imaging modality in clinical practice, and it is worthy of further exploration [31,32].

### 3.1. CT-Guided Contouring and Planning

Several studies have conducted comparative assessments of dimensions between delineations based on CT and MRI, demonstrating the practicality of contouring target volumes and OARs using CT scans. With the aid of diagnostic MRI without applicators in situ, CTV_HR_ can be contoured by CT. It is generally accepted that CT-based tumor contours could significantly overestimate tumor width [33], and some have also concluded that CT could also underestimate tumor heights [34,35,36]. As a result, CT may overestimate tumor volumes. Multiple studies have demonstrated that due to the overestimation of tumor volume by CT, the volume treated with a prescribed dose or higher (V100) is larger, which resulted in a lower CTV_HR_ D_90_ [34,36]. For the delineation of OARs, studies have mainly demonstrated that CT and MRI are comparable [34].

CT showed a poorer performance in the visualization of tumor and parametrial invasion than MRI [37]. Swanick et al. [38] found that agreement on CTV_HR_ spatial contours between CT and MRI was poor in patients with parametrial invasion >5 cm at the time of diagnosis, and with a high body mass index (BMI); thus, MRI guidance was recommended for such cases. Larger tumors and complex applicators were more likely to benefit from MRI [23]. In addition to the differences in tumor dimensions, different positions may also occur between CT- and MRI-based contours. The actual target volume may be located in a high-dose area or a dose-drop area, which will lead to the dose parameters guided by CT not truly representing the dose delivered to the actual volume [39]. This may explain the worse survival outcomes of CT-guided than MRI-guided therapy, without a significant difference in CTV_HR_ dose–volume parameters [40].

Based on the above findings, it is advisable to combine physical examination and pre-BT MRI to improve CT-guided delineations [20,34]. Furthermore, enhanced CT and/or ultrasound may also reduce the variations in and dose impacts of target delineation [41,42]. Several consensuses have been issued for CT-guided target delineation [43,44,45]. In 2021, the IBS-GEC ESTRO-ABS recommendation [45] defined CT-based CTV_HR_ under four different radiological environments and four different remission categories. CT or MR imaging at diagnosis, clinical examination and documentation, and CT imaging at BT with the applicator in situ were the minimum requirements for CT-based contours. In situations where only CT is available, two thirds of the uterine cavity is a surrogate for CTV_HR_ height to include most of the potential cranial tumor extension for advanced disease [45,46]. These guidelines are valuable for improving the consistency and quality of CT-guided BT, especially in resource-constrained settings. Nevertheless, further validation is required to assess their reproducibility and feasibility.

### 3.2. Clinical Outcomes

A great number of studies have published clinical results of CT-based BT (Table 1). Cho et al. [47] retrospectively analyzed 128 patients with FIGO stage I-II disease treated with IC. The overall LC, progression-free survival (PFS), and OS were 96%, 88%, and 88% at 2 years, respectively, and no isolated pelvic local recurrence was observed. They suggested that CT-based therapy may be considered for stage I and II cervical cancer or near-complete response after undergoing CCRT if MRI cannot be routinely used. Chan et al. [48] reported the results of follow-up over 4 years, with 25.9% receiving at least one IS, reporting 5-year LC 90.7%, DFS 80.0% and OS 87.2%. This was consistent with the previously mentioned target volume study, wherein the median volume of the CTV_HR_ delineated under CT guidance was larger compared with similar studies based on MRI, which led to a lower CTV_HR_ D_90_. There are currently few data to directly compare CT- and MRI-guided planning strategies. D’Cunha et al. [49] found that CTV_HR_ D_90_ and OARs’ D_2cc_ showed consistency between MRI- and CT-based BT. In the MRI group, 5-year OS was improved compared to the CT group (68.7% vs. 63.5%), as well as 5-year PFS (58.8% vs. 51.5%). However, upon univariate analysis these improvements were not significant. In another single-institutional study, no statistically significant differences were observed in CTV_HR_ EQD2 (equivalent dose in 2Gy/f) and OARs’ D_2cc_. But MR-guided ISBT was associated with better OS than CT-guided ISBT. (2y OS: 84% and 56% *p* = 0.036) [40]. Large-scale studies on patients with large volumes or receiving ISBT may support improved clinical outcomes with MRI. 

**Table 1:** Major studies that published clinical outcomes 3D image guidance modalities other than MRI in BT of LACC.

	Imaging Modality for Planning	IC or IC/IS	Number of Patients	FIGO Stage	Median Follow-Up (m)	Local Control (%)		Disease-Free Survival (%)		Overall Survival (%)		Toxicity (%)	
						3 y	5 y	3 y	5 y	3 y	5 y	Any	≥G3
Mesko et al. [50] 2015	CT	IS	31	IB–IVB	19	90 *		55 *		61 *			
Cho et al. [47] 2016	CT	IC	128	IA–IIB	30	96 *		88 *^,a^		88 *			16 ^c^, 2 ^d^
Ohno et al. [51] 2017	CT	IC, IC/ IS (17.5%)	80	I–IV	60		94		90 ^b^		86		3.8 (GI) ^d^ 3.8 (GU) ^d^
Kawashima et al. [52] 2019	CT	IC	84	IB–IVA	36	89		81 ^a^		94		28.8 (GI)	5 (GI)
Chan et al. [48] 2022	CT	IC, IC/ IS (25.9%)	135	IB–IVA	54		90.7		65.2		87.2	11.9	9.7
Xiu et al. [53] 2022	CT	IC, IC/ IS (31.3%)	211	IB2–IIIB	69		89		67		78	25.1(GI) 1.9 (GU)	12.3 (GI)
Uezono et al. [54] 2022	CT	IC, IC/ IS (27%)	171	IB–IV	33	86				75			5, 4 ^c^ 6 (GI) ^d^, 2.9 (GU) ^d^
Beriwal et al. [22] 2011	F1 MRI + serial CT	IC	44	IB–IIIB	8	88 *				86 *			0
Choong et al. [55] 2015	F1 HYBRID + serial CT	IC	49	IB–IVA	47	92.6		78.8 ^a^		77.7			12
Van Dyk et al. [56] 2016	single MR + serial US	IC	191	II–VB	60	86	86			75	63		6
Tharavichitkul et al. [57] 2018	TAUS	IC	92	IB–III	41	85.9		76.1		89.1			
Tharavichitkul et al. [58] 2022	CT (50.5%) TAUS (49.5%)	IC, IC/ IS (9.2%)	295	I–IV	48		89.5 ^#^		74.9 ^a,#^		69.1 ^#^		1.7 (≥G2 GU) 4.1 (≥G2 GI)
Kim et al. [59] 2022	PET/CT	IC	151	I–IV	57		89.2		64.1		76		8.6 ^c^, 7.3 ^d^

* 2-year; ^#^ 4-year. ^a^ Progression-free survival, ^b^ Pelvic progression-free survival. ^c^ Acute toxicity, ^d^ Late toxicity. IC = Intracavitary; IS = Interstitial; TAUS = Transabdominal ultrasound; GI = gastrointestinal; GU = genitourinary.

## 4. CT/MRI-Guided BT: Prominent Combination

Given the ongoing challenges in accurately defining target volumes using CT scanning alone, numerous medical institutions have attempted to combine CT and MRI modalities in guiding BT procedures. This integration aims to leverage the practicality of CT scans while harnessing the superior precision of MRI.

In the previously proposed method by Beriwal et al. [22], MRI images obtained before or at the first fraction (F1) of BT served as reference images for target delineation and dose optimization, without the need for image fusion [22]. (Table 2). While assessment of dose analysis and early clinical outcomes supported the feasibility of this approach, it placed a significant reliance on the expertise of the medical personnel involved. The introduction of image fusion techniques proved advantageous in achieving a more precise definition of the target volume. Various CT/MRI fusion-based methods have been employed (Table 2).

**Table 2:** Characterizations of different CT/MRI combined BT approaches.

Publication, Year	Institution	CT/MRI Combination	Characterization of Workflow	Advantages and/or Limitations
Beriwal et al. [22] 2011	Pittsburgh Cancer Institute	non-image fusion	MRI image at F1 BT as a reference	First report of a combined approach Highly dependent on the observers’ expertise
Nesvacil et al. [23] 2013	Vienna group	MRI at F1	CTV_HR_ delineated on MRI at F1 BT For subsequent fractions: MRI/CT fusion through applicator-based registration.	Requiring consistency in applicators Large deviations in IS and large tumors Compatible facilities are still necessary
Trifiletti et al. [60] 2015	Virginia University	asynchronous MRI image	A Smit sleeve is sutured and fixed to the external Os of the cervix, and then the applicator is inserted. CT images and CT-based planning obtained for F1 BT. After F1 treatment, the applicator and packing gauze are removed while the Smit sleeve remains in place. Then, MRI images are obtained, and GTV volume is defined on MRI images. For subsequent fractions: MRI/CT fusion uses rigid registration based on a Smit sleeve.	Accessible for centers without MRI-compatible applicators Retaining the logistical advantages of a CT-based workflow (familiarity, speed, and planning) Additional clinical visits and sutures
Choong et al. [55] 2015	St James’s University	F1 hybrid and F2-3 CT only	MRI and CT images with applications in situ at F1. A standard treatment plan is conducted (7 Gy prescribed to Point A) Post-plan created based on hybrid images CTV_HR_ transferred to F2-3 CT images using rigid registration based on applicators	F1 planning is not optimized Not applicable for bulky disease or IS

One of the most commonly utilized techniques was the approach proposed by the Vienna group, which employed MRI only for the first time of application [22]. The findings indicated that for small tumors, the MRI/CT plan exhibited a high degree of comparability with the MRI plan. Nevertheless, some limitations are inevitable. First, it requires consistency in both the model and positioning of the applicator during each fraction. Second, when using interstitial needles or in large tumors, the anatomical relationship changes greatly, resulting in large deviations in planning. Third, at least one MRI scan is needed, which is still not applicable in centers lacking MRI-compatible facilities.

Trifiletti et al. [60] applied the method of asynchronous MRI image fusion, in which MRI images without applicators in situ are fused to CT images based on a Smit sleeve. This approach could incorporate MRI-based target definition while retaining the advantages of a CT-based workflow. Dose analysis [61] showed that MRI fusion allowed the dose distribution to be adjusted according to individual anatomical characteristics, thus reducing excessive or insufficient coverage. Another study conducted by Choong et al. [55] obtained both MRI and CT with applicators in situ at F1, and subsequently registered these images in the follow-up fraction CT images, yielding promising results. When comparing this hybrid approach (n = 49) with the three-fraction MRI-guided approach (n = 27), the CTV_HR_ D_90_ and rectum/small bowel D_2cc_ were similar. As for clinical outcomes at 3 years, the local control hybrid group showed a comparable LC (92.6% and 92.2%, *p* > 0.05) and better OS (77.7% vs. 69.6%, *p* < 0.05) than the MRI group. But selection bias remained in this study, and a prospective study will be required.

In summary, the combined CT/MRI-guided BT approach is considered a cost-effective and highly accessible option, holding promise for the expansion of IGABT in LACC. A few clinical outcomes have been reported to support the combined modes. (Table 1) Nevertheless, certain limitations persist in the nature of this combined approach. Notably, the inability to observe tumor shrinkage throughout the treatment course using a single MRI underscores the importance of the physician’s comprehensive consideration of each patient’s clinical information.

## 5. PET-Guided BT: On the Way

FDG-PET is a functional imaging modality that is sensitive to distant metastasis and nodal diseases, and it has been used for staging the diagnosis of tumors and guiding radiotherapy intensity [62,63,64]. The investigation of PET-guided BT for cervical cancer aims to enhance the assessment of tumor regression following external beam irradiation and compensate for the suboptimal imaging quality of CT [65]. Lin et al. [66] revealed that PET-optimized plans had better dose coverage on the GTV than 2D plans based on point A, without increasing doses to rectal and bladder reference points. In this study, GTV was defined as the range of FDG-avid uptake on PET scans, identified by a 40% peak tumor intensity based on an early study [67]. The application of sequential PET could also help to better evaluate the response of the tumor during external radiation, and contribute to adaptive radiotherapy [68].

Nam et al. [65] first proposed the concept and procedural details of FDG-PET/CT-guided BT for cervical cancer. In this approach, target volumes were meticulously delineated based on the fusion of PET and CT images. GTV was identified as the enhancing area by adjusting the window and level to a reasonable value, guided by visual assessment in consultation with a nuclear medicine physician. CTV encompassed a 1 cm expansion towards the soft tissue mass boundaries of the uterus, cervix, and vagina and microscopic residual disease, which was based on baseline MRI and pelvic examination upon IGBT planning. PET-guided BT indicated a higher D_90_ of CTV than point A prescriptions, and long-term outcomes of a 5-year LC rate of 89% in comparison to other MRI-guided BTs [59]. Nonetheless, numerous challenges persist, including the absence of a definitive threshold for standardized uptake values (SUVs), substantial uncertainties at both intra- and inter-observer levels, and the necessity for further consensus in this field. Moreover, the burdensome procedure of radiation protection, scan intervals, and the need for continuous bladder irrigation may be barriers to its broader utilization.

Furthermore, a great number of studies have also concentrated on the predictive value of PET, and found that pretreatment values or relative changes in PET parameters [69], such as SUVmax and metabolic tumor volume (MTV), were associated with treatment responses and long-term outcomes [70]. Lucia et al. found that the high-uptake volume on baseline PET/CT was correlated with the site of recurrence, and suboptimal dosage coverage of the area could be associated with a higher risk of recurrence [71,72]. The investigation of the dose–effect relationship within these PET-based biological target volumes remains a crucial area of research. It holds the potential to delineate novel target volumes and dose prescriptions that may enhance treatment precision and efficacy.

## 6. US-Guided BT: Promising and Low-Cost

The exploration of US-guided BT is gaining prominence as an alternative approach in the treatment of cervical cancer. This is primarily attributed to its accessibility and invaluable real-time feedback. The assessment can be performed via transabdominal ultrasound (TAUS) and transrectal ultrasound (TRUS).

There is promising feasibility in employing US for contouring and optimizing treatment planning, primarily due to its outstanding soft-tissue resolution. The workflow can be guided solely by US or in conjunction with other imaging modalities (Table 3).

**Table 3:** Characterizations of different US-guided BT planning approaches.

Publication, Year	Institution	US-Guided BT Planning	Characterization of Workflow	Perspectives
Nesvacil et al. [73] 2016	Vienna group	TRUS/CT	Continuous 3D image acquisition by TRUS performed before (TRUS_preBT_) and after insertion (TRUS_BT_) of the applicator. Contouring CTV_HR_ on reconstructed transversal planes of TRUS_BT_ with TRUS_preBT_ also considered. TRUS/CT image fusion by rigid registration. CTV_HR_ transferred to CT and OARs delineated directly on CT.	High agreement with MRI Limited FOV and acoustic shadowing Still requires technical improvements for precise applicator reconstruction
Van Dyk et al. [74] 2015	Peter MacCallum Cancer Centre	single MR + serial TAUS	TAUS images obtained at F1; cervical and uterine widths measured on the transverse images. Only longitudinal images are imported to the TPS system to generate a US plan. MRI scanned with the applicator in situ after the treatment; 3D distribution of the US planning dose calculated on the MRI used as a guide for the optimization of the second ultrasound plan. For the subsequent fraction: if the position and measurements of the cervix and uterus are near to the previous fraction, a new plan can be implemented.	US used for both planning and verification of placement and dose coverage Not possible to evaluate volumetric dose of OARS
Tharavichitkul et al. [75] 2015	Chiang Mai University	TAUS/orthogonal radiographs	TAUS-guided tandem placement; relationship between tandem and the uterus were evaluated. Eight cervical points were defined by measuring distances from tandem to the uterine wall by TAUS in sagittal view and then marked on the orthogonal images. Dose optimization guided by Point A, ICRU reference points and eight cervical points.	US used for both planning and placement Not allowing full volumetric analysis of dose coverage Unable to evaluate the residual tumor or the vaginal extension Cannot accumulate doses to sigmoid colon and vagina

Schmid et al. [73,76] found that TRUS exhibited a strong agreement with MRI when assessing the CTV_HR_ width, accompanied by a slight underestimation of CTV_HR_ thickness. This underscores its potential for accurately depicting the treatment target. The Vienna group, recognizing the complementary benefits of TRUS and CT, recommended the fusion of TRUS and CT images while the applicator was in place. This approach was found to mitigate the overestimation of CTV_HR_ volume of CT alone, and yielded planning outcomes closely associated with MRI-based planning [77]. In follow-up studies, they developed an optical tracking device as a reference structure to overcome limitations in anterior and cranial borders of CTV_HR_ due to the limited probe length and its acoustic shadowing [78]. Swamidas et al. [41] also reported no statistically significant differences in CTV_HR_ and OARs between TRUS/CT- and MRI-based planning in 21 patients who received IC/IS or IC. However, the dosimetric and clinical outcome impacts of TRUS-guided BT in a large sample remain to be reported.

TAUS is also an option for BT planning. With a larger field of view (FOV) than TRUS, TAUS can clearly display the entire applicator and has a high consistency with MRI in defining CTV [79]. Van Dyk et al. [80] first reported dosimetric agreement between TAUS-based planning and 2D MRI imaging in doses delivered to target volume, the rectal point, and vaginal mucosa. They attempted to use single MRI combined with serial TAUS [74]. In this procedure, US was utilized to both generate a plan and to verify the placement and dose coverage. This practice has reported very low rates of ≥3 and adverse events, demonstrating the safety of the procedure, and further attempts will be made to apply hybrid applicators to improve target coverage [56]. Tharavichitkul et al. [58,75] reported their experiences and excellent outcomes of dose planning using TAUS and orthogonal radiographs. Eight cervical points were defined by TAUS and marked on the orthogonal images to serve as reference points to guide the dose optimization. In their report of 295 patients over ten years who underwent CT- or TAUS-guided BT, there were no significant differences between the two modalities in terms of local control and survival [58]. 

Briefly, US is an effective and portable method for institutions and regions with limited access to serial MRI guidance. The exceptional soft tissue resolution of US enables physicians to utilize it as a standalone tool or in conjunction with CT or MRI for treatment planning and generation. However, it is imperative for the medical community to establish operational consensus and drive technological advancements to address the inherent limitations associated with operator dependence, restricted field of view, and acoustic shadowing.

## 7. Emerging Techniques in Image Guidance

### 7.1. US-Guided Applicator Implantation

US was widely utilized to guide tandem implants to avoid uterine perforation and improve the accuracy of placement. The conventional blind insertion of tandems carried a heightened risk of perforation, particularly among elderly patients with varying cervical anatomical characteristics, stenosis of the cervical Os, and increased extent of disease [81]. Suboptimal placement of applicators could also negatively affect local control and survival [82]. A meta-analysis that included 12 studies with 1757 inserts and a phase III randomized trial demonstrated that US guidance could result in an almost 10-fold reduction in the risk of perforation per insertion [83,84]. Experiences have also been documented for patients encountering challenges in applicator placement [85,86]. The utilization of intraoperative ultrasound serves as a preventive measure against inadvertent insertion into false passages in a necrotic or fibrotic cervix or within the myometrium. It facilitates cervical manipulation and the selection of an appropriate tandem curvature [87]. In light of the aforementioned considerations, the American Society for Radiation Oncology (ASTRO) recommended real-time TAUS or TRUS guidance as the gold standard to assist tandem insertion to prevent perforation [88]. In practice, it is crucial to obtain simultaneous US visualization of the cervix and the tandem tip while it is inserted [89].

In recent studies, the feasibility of real-time US guidance of interstitial needles has been explored to adjust the direction and depth of needle insertion. Knoth et al. [90] studied the visualization of needles in TRUS, and found that most of the interstitial needles were visible. Insertion directions significantly affected visualization. Needles aligned parallel to the uterine axis, typically located within the cervix and/or tumor, demonstrated superior signal contrast and enhanced visibility compared with needles positioned within the parametrial space. Although the needle margins were blurred, quantitative analysis showed a high correlation between TRUS and MRI, with 89% within 3 mm. Lin et al. [91] also showed that TRUS had a high consistency with MRI in needle positioning. Rodgers et al. [92] invented a system which could reconstruct the 2D frames into a 3D TRUS image in real-time, and found 79% of tips were identifiable in vivo. Shadowing artifacts were the main obscuration.

Furthermore, it is anticipated that active tracking devices, such as electromagnetic tracking (EMT), can be integrated with US to enable real-time guidance and facilitate the digitalization of interstitial needles with greater accuracy and precision. Yang et al. [93] developed an intraoperative guidance system in which electromagnetic sensors attached to the needles enabled tracking needle tips and localizing the needle trajectories in space. Another sensor attached to the ultrasound probe registered 2D ultrasound with preoperative MRI. The probe calibration algorithm was used to obtain the relative coordinates of the current ultrasound image on the spatial position. The system provided a robust solution to realize the combination of high-precision visualization and real-time image guidance. The team then applied the procedure in a CT-based context and also observed high accuracy [94].

### 7.2. MRI-Guided Applicator Implantation

MRI is also under investigation for intraoperative interstitial implantation guidance to achieve more accurate positioning. According to how the interstitial catheters are visible in MRI, the approaches are separated into passive and active tracking [95]. The former is achieved depending on different contrasts between needles and surrounding tissues, while the latter relies on modern specific sensors to send signals of location and orientation. Viswanathan et al. [96] first reported a prospective clinical trial of MRI passive tracking in gynecologic ISBT. As the needle was inserted, real-time images were viewed with either fast T2 sagittal or axial images displayed on the MR screens above the operating area. The results demonstrated MRI-guided insertion contributed to proper placement and low morbidity. In Brigham and Women’s Hospital, an active MR tracking (MRTR) system was designed and evaluated [97,98]. The system could provide accurate catheter tip localization and catheter digitization by means of a stylet with microcoils. Moreover, the active tracking system presented a faster tracking update speed and the possibility of eliminating the need for a manual reconstruction process. As of now, the clinical assessment of MRI-guided real-time guidance is urgently lacking. Integration into the current workflow must be accompanied by sufficient evidence and standardized safety tests with great forethought.

### 7.3. 3D Printing Personalized Applicators and Implant Templates

Traditional freehand needle placement requires more technical skills than ICBT and relies heavily on experienced operators. To obtain higher dose coverage, it is necessary to repeatedly adjust the insertion angles and depths according to the post-implantation CT/MRI scanning, which may increase the risk of patient injuries. In addition, securing needles is difficult during patient transduction, leading to uncertainty in dosimetry. These disadvantages limit the use of ISBT. First proposed in the 1980s, 3D printing is a technology that creates entities by printing layer by layer. With the help of 3D-printing technology, individualized applicators and interstitial implant templates of specific shapes can be prepared according to the individual anatomical structure to help optimize the needle path, achieve accurate dose coverage, and improve security and efficiency [99,100]. Jiang et al. [101] summarized the advantages of the 3D template as follows: it can decrease setup and mobilization errors, and minimize differences between therapists. Moreover, MRI can be used in preplanning for better lesion definition and treatment quality control.

Lindegaard et al. [102] developed a 3D-printed tandem needle template (3DP TNT) in place of the ring channel of the T&R applicator. The template had eight guiding holes to provide precise and repeatable guidance for needles. The device helps treat nulliparous or elderly patients with vaginal stenosis who cannot tolerate the ring mold applicator, and may reduce vaginal mucosal complications. Recently, they published the long-term outcomes of routine clinical work using 3DP TNT, and implemented EMBRACE II planning aims and DVH constraints in 101 patients [103]. The study showed 3DP TNT was able to achieve an excellent mean CTV_HR_ D_90_ of 93.4 Gy, without exceeding EMBRACE II OAR dose constraints in infiltrative tumors and difficult anatomical conditions. Despite a high proportion of stage III/IV disease and a high tumor load, the 3-year LC and OS values were 85% and 63%, and ≥G3 side effects’ incidence was in line with retroEMBRACE and EMBRACE I. Marar et al. [104] designed a tandem anchored radially guiding interstitial template (TARGIT), which was attached to the tandem in place of a standard flange to guide four to six interstitial needles. The results demonstrated that compared with freehand implantation, TARGIT significantly improved the CTV_HR_ V_100%_ of tumors, especially in large tumors, and only slightly increased the operation time (an average of 6 min). Serban et al. [105] used a 3D-printed vaginal IC applicator as a template to guide parallel and oblique needles and achieved an HR CTV D_90_ of 92.5 Gy, 70% of which reached the hard dose limit of EMBRACE II. However, reports of clinical outcomes from 3D printing applicators are still scarce, and high-quality evidence is still needed to confirm the effectiveness of this application. Future research will also concentrate on the automatic generation of virtual stitches, automatic inverse optimization planning, and generation of personalized applicators [106,107]. User-friendly software should also be developed to avoid problems during data conversion. In addition, 3D printing-related norms and standards will also be formulated.

## 8. Conclusions

The transition to 3D-IGABT has been validated to improve survival outcomes while simultaneously reducing treatment-related toxicities. Among the available imaging modalities for IGABT, MRI stands out as the optimal option for contouring and planning due to its superior ability to visualize both tumor and normal tissues. Nevertheless, there is noticeable promise in exploring alternative imaging modalities, such as CT, PET, US, and their combinations. CT is the most wildly applied modality especially in developing countries, and it is considered to be combined with MRI or US to improve its contouring precision. There are also several preclinical and early-phase studies reporting PET- and US-guided BT. However, it is imperative that these alternatives undergo rigorous clinical evaluation to assess their efficacy. Emerging techniques for accurate tandem or interstitial needle guidance mainly include real-time guidance devices and 3D-printing applicators and implant templates. Real-time US guidance is recommended as the gold standard to assist tandem insertion. Other techniques, like real-time MRI-guidance and 3D-printing applicators and templates are under investigation. These emerging techniques will contribute to IGABT safety and tumor dose escalation. The future of BT for LACC is expected to progress towards personalized dose prescriptions and the refinement of more effective image-guided procedures. Moreover, achieving operational consensus among medical practitioners and driving technological advancements to mitigate the inherent limitations associated with different imaging modes remain essential.
